# Silicon availability modifies nutrient use efficiency and content, C:N:P stoichiometry, and productivity of winter wheat (*Triticum aestivum* L.)

**DOI:** 10.1038/srep40829

**Published:** 2017-01-17

**Authors:** Silke Neu, Jörg Schaller, E. Gert Dudel

**Affiliations:** 1 Institute of Plant and Wood Chemistry, Technische Universität Dresden, Pienner Straße 19, 01737 Tharandt, Germany; 2 Environmental Geochemistry, Bayreuth Center for Ecology and Environmental Research (BayCEER), University Bayreuth, Universitätsstraße 30, 95447 Bayreuth, Germany; 3 Institute of General Ecology and Environmental Protection, Technische Universität Dresden, Pienner Straße 7, 01737 Tharandt, Germany

## Abstract

Silicon (Si) is known as beneficial element for graminaceous plants. The importance of Si for plant functioning of cereals was recently emphasized. However, about the effect of Si availability on biomass production, grain yield, nutrient status and nutrient use efficiency for wheat (*Triticum aestivum* L.), as one of the most important crop plants worldwide, less is known so far. Consequently, we assessed the effect of a broad range of supply levels of amorphous SiO_2_ on wheat plant performance. Our results revealed that Si is readily taken up and accumulated basically in aboveground vegetative organs. Carbon (C) and phosphorus (P) status of plants were altered in response to varying Si supply. In bulk straw biomass C concentration decreased with increasing Si supply, while P concentration increased from slight limitation towards optimal nutrition. Thereby, aboveground biomass production increased at low to medium supply levels of silica whereas grain yield increased at medium supply level only. Nutrient use efficiency was improved by Si insofar that biomass production was enhanced at constant nitrogen (N) status of substrate and plants. Consequently, our findings imply fundamental influences of Si on C turnover, P availability and nitrogen use efficiency for wheat as a major staple crop.

After oxygen silicon (Si) is the most abundant element in the Earth’s crust^1^. A large turnover of Si was found to occur in terrestrial and semi aquatic ecosystems, with plants and plant litter decomposition being the driving force^2^,^3^,^4^. In plants a variety of functions are attributed to Si, especially to its amorphous forms, like promotion of biomass production or defense against various forms of biotic and abiotic stress^5^,^6^,^7^. Generally, graminaceous plants such as grasses are known to accumulate high amounts of Si in their aboveground tissues when compared to other plants^8^,^9^,^10^,^11^. Uptake mechanisms for Si and the formation of intracellular opal phytoliths with beneficial effects on plant stiffness are well described^12^,^13^. Low energy costs during the incorporation of Si into plant tissue relative to organic compounds^14^ already raised presumptions whether Si could act as a substitute for carbon (C) compounds in the plant. This was already proven with subsequent effects on the stoichiometry of C, nitrogen (N) and phosphorus (P)^6^. Recent studies could verify strong antagonistic correlations between Si supply and cellulose content in macrophytes^15^. For common reed a reduction of cellulose content in tissues with stabilization function could be observed due to Si surplus as well as a decline of C concentration in leaves^6^,^16^. Antagonistic correlations were also found between Si and N for emergent wetland plants^17^ and wheat^18^. In contrast Si was shown to promote P status and biomass production of common reed at moderate supply levels while high supply levels induced a negative effect on both parameters^6^. These findings point to a potential of Si to affect P availability and enhance the nutrient use efficiency, expressed as biomass produced per unit of a certain essential nutrient accumulated^19^ with regard to N. Thereby Si fertilization might reduce the need for mineral P and N fertilization in agricultural soils for crop plant production. Besides perennial species of the *Poacae* family, which are constituting large proportions of the world’s pasture areas, cereals are playing an important role in terrestrial Si cycling^20^. They are the most prevalent group of crops across the world, with wheat (*Triticum aestivum* L.) being one of the most widely grown crops, covering more than 20% of the global cultivated area^21^,^22^. Even though it is not generally regarded as an essential element for plant nutrition, Si is known to accumulate in concentrations of up to 4% (w/w) in wheat straw, which is comparable to essential plant macronutrients^18^. Si shortage was found to cause physical abnormalities in wheat plants in hydroponic cultures^23^. Another study in hydroponic culture showed that the presence of Si mitigates salinity stress and related adverse effects on dry matter yield and chlorophyll content in wheat^24^. Coinciding with these findings Si is supposed to be involved in metabolic and/or physiological mechanisms of wheat under drought stress, which is alleviated by the presence of high Si availability in soils^25^. However, nothing is known so far about the effect of Si availability on biomass production, nutrient stoichiometry and nutrient use efficiency of wheat.

Consequently, the effect of Si supply on C:N:P stoichiometry and plant growth of wheat as a major staple crop was assessed with implications for nutrition, fertilization and carbon turnover in agricultural soils. We hypothesize that high supply of plant available Si will (i) enhance plant P status with subsequent increase of biomass production and yield, (ii) improve the nutrient use efficiency and (iii) strongly affect the nutrient stoichiometry of wheat plants.

## Results

### Biomass production

**Figure 1:**
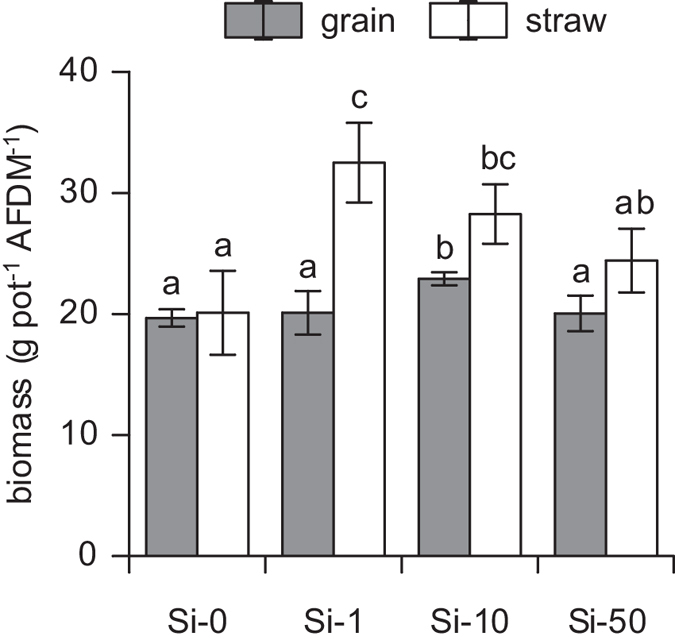
Grain and straw biomass of *Triticum aestivum* L. cv. Akteur expressed as ash free dry mass (AFDM) per pot without application of SiO_2_ to the substrate (Si-0) and with application of 1 g (Si-1), 10 g (Si-10) and 50 g (Si-50) of SiO_2_. Values are means ± SD (n = 4); different letters indicate significant differences between treatments at *p* < 0.05.

The production of aboveground biomass was clearly enhanced in pots that received 1 g SiO_2_ pot^−1^ (Si-1) and 10 g SiO_2_ pot^−1^ (Si-10), both differing significantly from the control (Si-0). Si-1 treatment also differed significantly from highest supply level of 50 g SiO_2_ pot^−1^ (Si-50) (Fig. 1). Thereby, the highest increase of biomass was found at low supply levels and gradually decreased with increasing Si supply. The rise in biomass production, proving hypothesis (i), mainly based upon a substantial increase of straw (culms and leaves) biomass, partly due to insignificantly higher shoot lengths, while grain yield only increased significantly when treated with Si-10 (Fig. 1).

### Concentration and stoichiometry of silicon and nutrients

Increasing supply of silica consistently increased Si concentration in belowground and aboveground plant tissue, except for wheat grain where it was kept at a constant level (Supplementary Table S1). Increases of Si concentration were found to be highly significant (*p* < 0.001) for vegetative tissue in case of medium (Si-10) and high application rates (Si-50) and for root tissue only in the latter case. Regarding the distribution within the plant Si was accumulated in decreasing order in leaf blades (flag leaf > subjacent leaf levels) ≥ leaf sheaths (flag leaf ~ subjacent leaf levels) > culm > root > grain.

**Figure 2:**
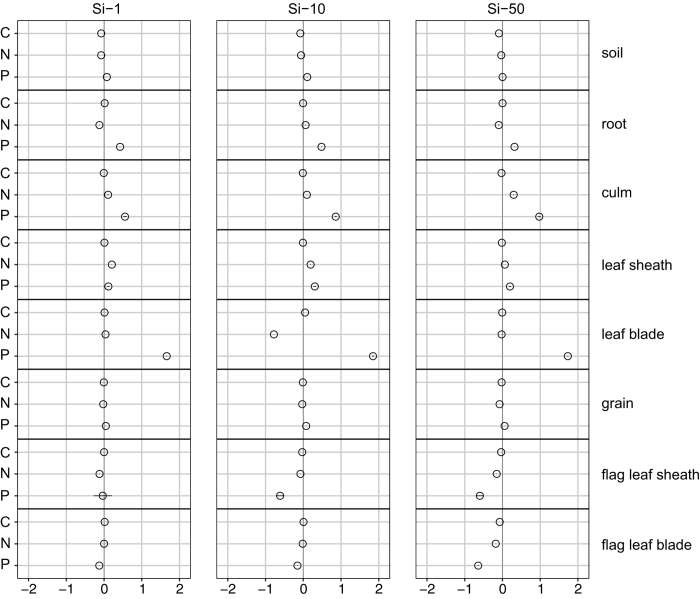
Changes [ln (x-Si-L/x-Si-0) ± ln (SD-Si-L/SD-Si-0), where x is the mean value of the observed variable and L the level of Si addition; n = 4] in molar carbon, nitrogen and phosphorus concentration of analyzed compartments when treated with differing levels of silica supply.

Carbon concentration tended to decrease with increasing Si concentration. This decrease was significant at medium (*p* < 0.05) to high (*p* < 0.001) supply levels of Si (Si-10, Si-50) in vegetative plant parts, particularly in culms and leaf sheaths, and in flag leaf sheaths (*p* < 0.05) and flag leaf blades (*p* < 0.001) for Si-50 only (Fig. 2, Supplementary Table S1 and S2). Substrate sampled along with the harvest of plant biomass also showed a significant (*p* < 0.001) decrease of C in Si-10 and Si-50 treatments when compared to Si-0 while no concentration gradient was found for root tissue.

Nitrogen concentration only differed significantly between the treatments in case of leaf tissue (*p* < 0.05 for leaf sheaths and flag leaf blades and *p* < 0.001 for leaf blades). A pronounced reduction of N was found for flag leaf blades of Si-50 (Fig. 2, Supplementary Table S2), whereas in subjacent leaf sheaths N was enhanced at low (Si-1) and medium (Si-10) supply levels. Another exception presented leaf blades of subjacent leaf levels treated with Si-10 having drastically lowered N and enhanced C concentration when compared to all other variants, which was an isolated phenomenon.

**Figure 3:**
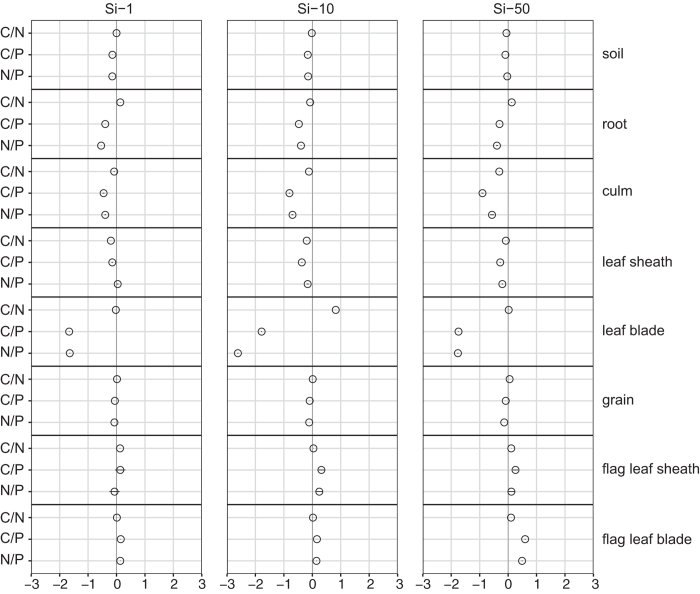
Changes [ln (x-Si-L/x-Si-0) ± ln (SD-Si-L/SD-Si-0), where x is the mean value of the observed variable and L the level of Si addition; n = 4] in molar element stoichiometry (C:N, C:P and N:P ratios) of analyzed compartments when treated with differing levels of silica supply.

Phosphorus status tended to increase with increasing silica supply. Generally, roots (*p* < 0.05), culms (*p* < 0.05) and leaf blades (*p* < 0.001) exhibited an increase of P status with increasing Si supply, while a slight but insignificant decrease was indicated for leaf tissue at the highest supply level Si-50. However, the results of flag leaves were contradictory in case of P as they generally showed a decrease of P status with increasing silica supply. Along with that C:P and N:P nutrient ratios were altered, proving hypothesis (iii), towards an increase with increasing silica supply in case of flag leaf blades (*p* < 0.05) (Fig. 3, Supplementary Table S2) but vice versa in leaf blades of subjacent leaf levels (*p* < 0.001) as well as in roots (*p* < 0.05), culms (*p* < 0.05) and for N:P also in wheat grain (*p* < 0.05) (Fig. 3, Supplementary Table S1). In the substrate both ratios were highest in Si-0 followed by Si-50 and similarly low in Si-1 and Si-10. The C:N nutrient ratio did not show a clear pattern. It tended to decrease with increasing silica supply in culms and similarly in leaf sheaths (*p* < 0.05), where it increased again at the highest supply level of Si-50. In leaf blades no gradient could be observed but the ratio for Si-10 was exceptionally high due to the previously mentioned low N concentrations. In flag leaf sheaths, flag leaf blades (*p* < 0.05) and grains (*p* < 0.05) the C:N ratio tended to increase with increasing supply of silica.

**Figure 4:**
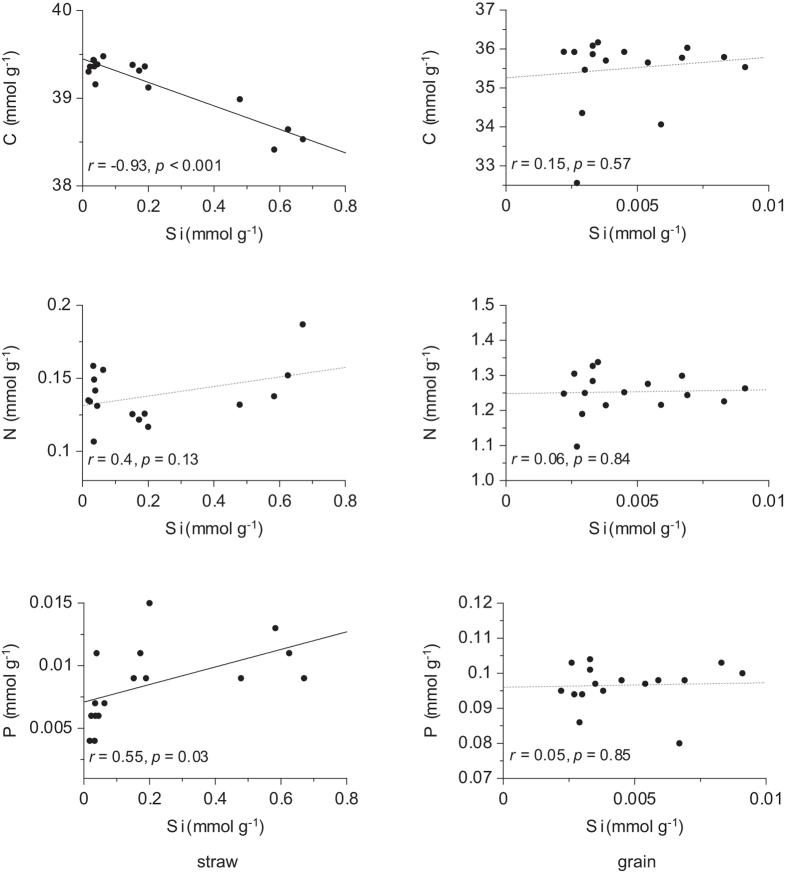
Linear regressions between silicon and nutrient concentration in straw and grain tissue of *Triticum aestivum* L. cv. Akteur. Pearson´s correlation coefficient (*r*) and the *p*-value are shown; significant correlations (*p* < 0.05) are indicated by black solid lines and non-significant correlations (*p* > 0.05) by grey dashed lines.

Linear regressions between Si and nutrients were conducted separately for straw and grain of wheat plants. For the latter no correlations were found. In contrast, for vegetative plant parts a strong and highly significant negative correlation was found between Si and C concentration. Also, a moderate but significantly positive correlation was found between Si and P, whereas N and Si were not significantly correlated (Fig. 4).

**Figure 5:**
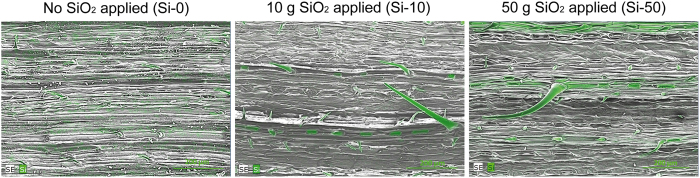
Distribution of silicon in flag leaf blades of *Triticum aestivum* L. cv. Akteur without (Si-0) and with exposition (Si-10, Si-50) to SiO_2_ applied to the substrate. Silicon appears in green color.

Investigation of flag leaves with scanning electron microscopy (SEM) revealed the formation of distinct trichomes (leaf epidermis hairs) and phytoliths (silica bodies within the epidermis) where Si was accumulated (Fig. 5) if applied to the substrate in medium (Si-10) and high (Si-50) concentration, which could not be observed in the control treatment (Si-0). The phytoliths and amorphous Si were distinctly arranged within the epidermal tissue.

## Discussion

Silicon, applied to the substrate in the form of an engineered nanomaterial (ENM) consisting of amorphous pyrogenic hydrophilic SiO_2_, was readily taken up by the studied wheat plants. The accumulation of Si was highest in vegetative tissue (leaf blades > leaf sheaths > culm) and lowest in grain followed by roots, increasing with increasing stomata density in the tissues. This is in accordance with previous studies where absorption of Si by roots of higher plants was shown to occur predominantly as silicic acid [Si(OH)_4_] and uptake was conducted actively, via specific transporters mediating xylem loading of Si, and passively with the transpiration pull. Thereby, wheat plants were shown to transfer approximately 90% of absorbed Si to the shoots whereas root concentration was maintained at relatively low levels^8^. Within photosynthetically active tissue Si was then polymerized and precipitated in the course of water loss driven by transpiration^1^,^8^,^13^. We found precipitation at medium (Si-10) and high (Si-50) levels to occur as phytoliths and further localized Si in strongly developed trichomes. Amorphous Si deposits^26^ appeared in case of Si-50 distinctly arranged between the previously mentioned structures. Typically Si is most enriched in oldest leaves, whereas we found most Si accumulated in flag leaf blades when compared to the bulk sample of subjacent leaf blades. This might be attributed to higher levels of transpiration driven water loss in flag leaves after ear emergence e.g. due to a higher exposition to wind and sunlight^27^. Investigations with a large quantity of barley varieties suggested that Si concentration in barley grain is under genetic control^28^. This might be the same for wheat grain and may explain that no changes in Si concentration were found despite markedly differing supply levels.

Our findings with regard to responses of plant C, N and P status to different levels of plant available Si are broadly in line with those obtained so far for wetland species^6^,^17^. For a variety of terrestrial plant species notably significant negative correlations between Si and C concentration as well as C-based defenses were found^29^. In this study also one member of the *Poaceae* family (*Entolasia stricta*) was included, but yet, these relations were not assessed for *Triticeae*. The highly significant negative correlation between Si and C in the *T. aestivum* cultivar of our study suggests that the incorporation of Si into tissue with stabilizing function or photosynthetically active tissue might serve as a proper surrogate for part of organic C compounds in cereals as well. Previous studies led to the conclusion that plant Si provides protection against mammal herbivory as well as chewing and sucking insects. This protection is driven by plant physical properties, as found in our study in the form of phytoliths or fortified trichomes^29^,^30^.

The concentration of N was not affected by plant Si in terms of the majority of plant compartments but leaf tissue. However, nutrient use efficiency was improved at whole-plant level by Si in such way that biomass production was enhanced at constant N status of substrate and plants, proving hypothesis (ii). Thereby, nitrogen use efficiency, if simply calculated as ratio of biomass production (g AFDM^−1^) to N uptake (g), increased from 102  ±  11 (Si-0) to 140 ± 13 (Si-1), 125 ± 4 (Si-10) and 126 ± 12 (Si-50) respectively. Yet, with respect to grain yield nitrogen use efficiency, as calculated using the formula [(plant N content at maturity/soil N supply) * (grain dry weight/plant N content at maturity)]^31^, was only positively affected in case of Si-10 (*p* < 0.01).

At moderate grain and relatively low straw P levels^32^,^33^,^34^ our results further revealed that the plant P status is altered as a function of silica supply, which supports recent findings about interferences between P metabolism of *Graminae* and Si accumulation^6^,^35^. This may be based upon competition between Si and P for binding sites on soil particles and subsequent increase of P in pore water and plant tissue of +Si treatments with highly soluble Si^36^ (see supplementary information of Seyfferth & Fendorf, 2012). In our study leaf blades responded with significant elevation of P status already at a low level of added silica (Si-1), while culms only responded at highest addition (Si-50). However, contradicting findings for flag leaf tissue hint at a translocation of P from flag leaf to grain or other tissue. Hence, an influence of plant available silica on nutrient stoichiometry is evident. Thereby, aboveground biomass production was negatively correlated with N:P ratio (*p* < 0.05) in aboveground plant tissue. The N:P ratios we found for Si-0 suggest a certain P limitation^37^. Therefore, without simultaneous increase of Si in plant tissue, biomass production was promoted already in case of Si-1 due to increased P status of plants. A further improvement of P nutrition within the optimal range might explain enhanced resource allocation towards reproductive organs^38^, at the expense of vegetative biomass, leading to significantly increased grain yield in Si-10. Consequently, the positive effect on biomass production in Si-1 and Si-10 has to be attributed basically to enhanced P availability and possibly further to a substitution of some parts of the carbon compounds (like cellulose) by silica, as found before^16^. The negative effect of Si at the highest supply level may however be explained by high Si levels within the plant possibly leading to negative effects on nutrient accumulation and biomass production, as found earlier^6^,^39^. Furthermore, despite decreasing C in straw of +Si treatments, biomass production was positively correlated with C:N ratio (*p* < 0.01), which is basically a function of altered harvest index (grain biomass/aboveground biomass) in +Si treatments leading to lowered N concentration in aboveground plant tissue.

In conclusion our results revealed that wheat plants cultivated under slightly P limiting conditions (i) increase biomass production of the whole plant at low (Si-1) and medium (Si-10) supply levels of silica and change resource allocation towards enhanced production of vegetative biomass (Si-1) or both vegetative and generative biomass in varying degrees (Si-10). This stimulation of plant growth may basically be attributed to improved P availability. Nevertheless the optimal range of silica supply seems to be reached at moderate application rates (Si-10), because both vegetative and generative biomass markedly decrease again at high supply level. Moreover, we conclude that Si (ii) improves nitrogen use efficiency under the experimental conditions given in this study and (iii) is involved in C and P metabolism with subsequent effects on nutrient stoichiometry of wheat as a major staple crop. Therefore, Si should be considered as soil amendment in agricultural soils deficient in plant available Si as a means of sustainable agriculture with respect to possible savings of scarce P resources (P fertilizer). For an assessment of beneficial ranges of Si supply for a certain agricultural crop or cultivar further research is needed including varying levels of nutrient supply.

## Methods

### Experimental set-up

In order to avoid elevated Si concentrations in the substrate pure peat (Florbest) and a garden substrate containing peat and natural clay minerals (Einheitserde Special) purchased from Einheitserdewerke Werkverband e.V. were mixed at a ratio of 1:1 (v/v). Prior to application of silicon the substrate was treated with steam. Silicon was added in the form of Aerosil^300^ (Evonik Industries AG, Essen, Germany), an ENM consisting of hydrophilic pyrogenic silicon dioxide (SiO_2_). The global ENM material flows are currently dominated by SiO_2_ being used in products of various economic sectors. Most of it is assumed to be disposed to landfills but a significant share is also expected to enter soil, water and atmosphere^40^. The used Si-ENM Aerosil^300^ has a weakly acidic pH (~4.7) and a specific surface area of 300 m^2^ g^−1^. It was applied in pulverized form as purchased from the company in concentrations of 0 (Si-0), 1 (Si-1), 10 (Si-10) and 50 (Si-50) g pot^−1^ in four replicates each, thoroughly homogenized with the substrate. Each 13 L pot was filled with 1 kg dry mass of the respective substrate variant. Commercial seeds of *Triticum aestivum* L. cv. Akteur were purchased from the plant breeding company IG Pflanzenzucht GmbH, Germany and sterilized by means of electron beam at Fraunhofer Institute for Organic Electronics, Electron Beam and Plasma Technology (FEP) in Dresden, Germany. During sowing of initially 22 seeds in October 2014, all seeds were inoculated with vesicular-arbuscular mycorrhizae (VAM) by adding 300 l/ha INOQ_Spezial_ (INOQ GmbH, Schneba, Germany). After germination the pots were arranged outside in a sand bed in order to prevent frost damage during wintertime^41^. The sand was carefully filled in the gaps between closely arranged pots, which were placed on mats. In doing so contact between substrate and sand was avoided. In early spring pots were randomly arranged in greenhouses with filtered air and ambient temperature. During that time the number of plants was reduced to 16 per pot. Daily watering with deionized (DI) water was conducted in order to maintain the moisture regime at 70% field capacity. Fertilization was conducted twice with the commercial fertilizer Hakaphos soft Plus from COMPO Expert GmbH with an N:P:K ratio of 1:0.4:1.7, a NO_3_:NH_4_ ratio of 54:46 and water soluble magnesium oxide (3%), boron (0.01%), copper (0.02%), iron (0.075%), manganese (0.05%), molybdenum (0.001%) and zinc (0.015%). The first application was done in early April with an amount corresponding to 80 kg N ha^−1^ and a second one at the beginning of May corresponding to 40 kg N ha^−1^. In early June a third N application was conducted with NH_4_NO_3_ corresponding to 20 kg N ha^−1^. The initial pH (H_2_O) of the substrate was 6.0, representing the lower range of optimal growth conditions for winter wheat. In the course of plant growth pH dropped to ~5.0 at harvest.

### Soil and Plant analysis

At full maturity of grains in July plant shoots were clipped 2 cm above the substrate surface and aboveground biomass was separated into corn, leaf blades, leaf sheaths and culms. Leaf blades and leaf sheaths of the flag leaf were additionally separated from those of the subjacent leaf levels. A representative part of the root biomass was isolated from the substrate by sieving and washed with DI water. All plant tissues were subsequently dried at 50 °C to constant weight and ground to <0.25 mm particle size. The substrate was air-dried and sieved through a 2 mm nylon mesh, finely ground to <150 μm and dried at 50 °C prior to analysis. For analysis of P the plant material was digested in a microwave digestion system (CEM Mars5, CEM Corporation, Matthews, NC, USA) at 180 °C with 3 ml HNO_3_ and 2 ml H_2_O_2_^42^. Soil material was digested with 9 ml HCl and 3 ml HNO_3_. Elemental concentrations of further nutrients, analyzed as described above unless otherwise specified, are provided in Supplementary Table S3 (soil) and Supplementary Figure S1 (grain and flag leaf blades). Extraction of Si was conducted by an alkaline method using 30 mg soil or plant material and 40 ml of 0.1 *M* Na_2_CO_3_ for 5 h at 85 °C in a shaking water bath^7^. The extraction solution was subsequently passed through a 0.45 μm syringe filter. All samples were analyzed by ICP-OES (IRIS Intrepid II XSP, Thermo Fischer scientific). Chemical blanks and standard reference material NCS 73349 were used for validation. The content of carbon (C) and nitrogen (N) were analyzed with an Elementar Vario EL III elemental analyzer (Hanau, Germany)^43^. All chemicals were of analytical grade. Parts of roots and flag leaf blades were analyzed with scanning electron microscopy^6^.

### Statistical analyses

Treatment effects on biomass production and concentration of Si, C, N and P in plant tissue were evaluated using one way analysis of variance (ANOVA). All data met assumptions of homogeneity of variance and normal distribution of residuals. Differences between the treatments were assessed using the Tukey-HSD *post hoc* test. Statistical analyses were performed using PASW statistics 21 (SPSS, Inc., Somers, NY, USA). Log response values for the figures were calculated by ln of mean values (Si-treatment/control) using the following formula^44^: ln (Ῡ_Si-treatment_/Ῡ_control_) ± (s^2^_Si-treatment_/n _Si-treatment_ Ῡ^2^_Si-treatment_) + (s^2^_control_/n_control_ Ῡ^2^_control_). If log response ratio is zero then the Si-treatment values do not differ from the control. If the log response ratio is below zero the Si-treatment values are lower than the control and if log response ratio is larger than zero the Si-treatment value is larger than the control.

## Additional Information

**How to cite this article**: Neu, S. *et al*. Silicon availability modifies nutrient use efficiency and content, C:N:P stoichiometry, and productivity of winter wheat (*Triticum aestivum* L.). *Sci. Rep.* **7**, 40829; doi: 10.1038/srep40829 (2017).

**Publisher's note:** Springer Nature remains neutral with regard to jurisdictional claims in published maps and institutional affiliations.

## Supplementary Material

Supplementary Information
