# Supplementary material for: Silicon availability modifies nutrient use efficiency and content, C:N:P stoichiometry, and productivity of winter wheat (Triticum aestivum L.)
Source: Sci Rep. 2017 Jan 17;7:40829. doi: 10.1038/srep40829 (PMC5240101; doi:10.1038/srep40829)

Silicon availability modifies nutrient use efficiency and content, C:N:P stoichiometry, and productivity of winter wheat (*Triticum aestivum* L.)

Silke Neu[1 *], Jörg Schaller[2], E. Gert Dudel[1,3]

[1]Institute of Plant and Wood Chemistry, Technische Universität Dresden, Pienner Straße 19, 01737 Tharandt, Germany

[2]Environmental Geochemistry, Bayreuth Center for Ecology and Environmental Research (BayCEER), University Bayreuth, Universitätsstraße 30, 95447 Bayreuth, Germany

[3]Institute of General Ecology and Environmental Protection, Technische Universität Dresden, Pienner Straße 7, 01737 Tharandt, Germany

Supplementary Information

Table S1 Variation in concentration of Si, nutrients and nutrient stoichiometry in substrate and plant tissue of *Triticum aestivum* L. cv. Akteur (mmol g^{-1} $AFDM^{-1}$). Values are means ± SD; different letters indicate significant differences (^{abc} for $p < 0.001$, ^{de} for $p < 0.05$).

		soil	root	culm	leaf sheath	leaf blade	grain
silicon	Si-0	0.01 ± 0.01 [a]	0.001 ± 0.001 [a]	0.01 ± 0.00 [a]	0.06 ± 0.01 [a]	0.11 ± 0.02 [a]	0.005 ± 0.002
	Si-1	0.08 ± 0.01 [a]	0.016 ± 0.003 [a]	0.03 ± 0.02 [ab]	0.13 ± 0.01 [a]	0.21 ± 0.02 [a]	0.003 ± 0.001
	Si-10	0.19 ± 0.01 [a]	0.060 ± 0.022 [a]	0.11 ± 0.01 [b]	0.56 ± 0.06 [b]	0.60 ± 0.08 [b]	0.006 ± 0.003
	Si-50	0.96 ± 0.19 [b]	0.172 ± 0.034 [b]	0.40 ± 0.05 [c]	1.52 ± 0.19 [c]	2.28 ± 0.15 [c]	0.005 ± 0.003
carbon	Si-0	40.1 ± 0.5 [b]	39.2 ± 0.6	39.7 ± 0.2 [b]	38.4 ± 0.1 [bc]	38.5 ± 0.2 [ab]	35.8 ± 0.3
	Si-1	37.3 ± 0.5 [ab]	39.9 ± 0.7	39.4 ± 0.2 [ab]	38.8 ± 0.1 [c]	39.0 ± 0.1 [b]	35.6 ± 0.8
	Si-10	37.2 ± 0.9 [a]	39.0 ± 0.4	39.3 ± 0.1 [ab]	38.2 ± 0.1 [ab]	40.4 ± 0.2 [c]	35.4 ± 0.9
	Si-50	36.6 ± 1.1 [a]	39.4 ± 0.2	38.7 ± 0.2 [a]	37.8 ± 0.1 [a]	38.3 ± 0.2 [a]	35.0 ± 1.6
nitrogen	Si-0	0.74 ± 0.02	0.55 ± 0.07	0.10 ± 0.01	0.17 ± 0.01 [d]	0.37 ± 0.02 [b]	1.29 ± 0.04
	Si-1	0.69 ± 0.01	0.49 ± 0.04	0.11 ± 0.03	0.20 ± 0.01 [e]	0.39 ± 0.02 [b]	1.26 ± 0.06
	Si-10	0.70 ± 0.01	0.59 ± 0.05	0.11 ± 0.00	0.20 ± 0.03 [e]	0.17 ± 0.01 [a]	1.26 ± 0.04
	Si-50	0.72 ± 0.04	0.50 ± 0.10	0.14 ± 0.02	0.18 ± 0.01 [de]	0.36 ± 0.02 [b]	1.20 ± 0.07
phosphorus	Si-0	0.012 ± 0.001	0.013 ± 0.001 [d]	0.003 ± 0.000 [d]	0.013 ± 0.004	0.009 ± 0.001 [a]	0.092 ± 0.008
	Si-1	0.013 ± 0.001	0.021 ± 0.002 [e]	0.005 ± 0.002 [de]	0.014 ± 0.002	0.049 ± 0.005 [b]	0.097 ± 0.008
	Si-10	0.014 ± 0.002	0.022 ± 0.003 [e]	0.006 ± 0.002 [de]	0.017 ± 0.002	0.059 ± 0.015 [b]	0.100 ± 0.002
	Si-50	0.012 ± 0.001	0.018 ± 0.002 [e]	0.007 ± 0.003 [e]	0.016 ± 0.001	0.053 ± 0.009 [b]	0.098 ± 0.004
C:N	Si-0	54 ± 1	72 ± 9	397 ± 42	233 ± 18 [e]	104 ± 4 [a]	27.7 ± 0.6 [d]
	Si-1	54 ± 1	82 ± 7	366 ± 88	192 ± 14 [d]	101 ± 5 [a]	28.2 ± 0.8 [de]
	Si-10	53 ± 1	67 ± 6	354 ± 8	192 ± 25 [d]	237 ± 11 [b]	28.1 ± 0.5 [de]
	Si-50	51 ± 4	81 ± 17	291 ± 44	215 ± 11 [de]	106 ± 7 [a]	29.3 ± 0.3 [e]
C:P	Si-0	3245 ± 223	2928 ± 105 [b]	15289 ± 1518 [e]	3216 ± 811	4248 ± 642 [b]	391 ± 37
	Si-1	2807 ± 262	1967 ± 263 [ab]	9693 ± 3468 [d]	2772 ± 474	805 ± 89 [a]	369 ± 24
	Si-10	2762 ± 527	1827 ± 304 [a]	6857 ± 2123 [d]	2225 ± 265	714 ± 155 [a]	355 ± 8
	Si-50	2951 ± 119	2166 ± 316 [ab]	6216 ± 2279 [d]	2442 ± 164	742 ± 110 [a]	359 ± 15
N:P	Si-0	60 ± 4	41 ± 4 [e]	39 ± 8 [e]	14 ± 4	41 ± 9 [b]	14.1 ± 1.4 [e]
	Si-1	52 ± 5	24 ± 3 [d]	26 ± 7 [de]	15 ± 3	8 ± 1 [a]	13.1 ± 0.6 [de]
	Si-10	52 ± 9	28 ± 6 [d]	19 ± 6 [d]	12 ± 3	3 ± 1 [a]	12.6 ± 0.2 [de]
	Si-50	58 ± 6	28 ± 9 [d]	22 ± 11 [de]	11 ± 1	7 ± 1 [a]	12.3 ± 0.6 [d]

Table S2 Variation in concentration of Si, nutrients and nutrient stoichiometry in flag leaf tissue of *Triticum aestivum* L. cv. Akteur (mmol g^{-1} $AFDM^{-1}$). Values are means ± SD; different letters indicate significant differences (^{abc} for $p < 0.001$, ^{de} for $p < 0.05$).

		flag leaf sheath		flag leaf blade	
silicon	Si-0	0.07 ± 0.01	a	0.15 ± 0.04	a
	Si-1	0.13 ± 0.01	a	0.28 ± 0.05	a
	Si-10	0.42 ± 0.04	b	0.74 ± 0.05	b
	Si-50	1.58 ± 0.04	c	3.65 ± 0.55	c
carbon	Si-0	39.1 ± 0.2	e	38.3 ± 0.4	b
	Si-1	39.2 ± 0.1	e	39.0 ± 0.6	b
	Si-10	38.1 ± 1.0	de	38.7 ± 0.1	b
	Si-50	37.8 ± 0.4	d	35.6 ± 0.4	a
nitrogen	Si-0	0.22 ± 0.03		0.38 ± 0.02	e
	Si-1	0.20 ± 0.02		0.39 ± 0.02	e
	Si-10	0.20 ± 0.01		0.38 ± 0.02	e
	Si-50	0.19 ± 0.01		0.32 ± 0.02	d
phosphorus	Si-0	0.02 ± 0.01		0.04 ± 0.003	b
	Si-1	0.02 ± 0.02		0.03 ± 0.004	ab
	Si-10	0.01 ± 0.00		0.03 ± 0.004	ab
	Si-50	0.01 ± 0.00		0.02 ± 0.004	a
C:N	Si-0	179 ± 22		100 ± 4	d
	Si-1	203 ± 25		101 ± 4	d
	Si-10	187 ± 15		102 ± 5	de
	Si-50	200 ± 8		111 ± 4	e
C:P	Si-0	2566 ± 1549		1100 ± 113	d
	Si-1	2924 ± 1767		1277 ± 158	d
	Si-10	3536 ± 744		1295 ± 149	d
	Si-50	3328 ± 212		2004 ± 469	e
N:P	Si-0	14.8 ± 9.8		11.0 ± 1.0	d
	Si-1	13.8 ± 7.3		12.6 ± 1.3	d
	Si-10	18.9 ± 3.9		12.7 ± 1.6	d
	Si-50	16.6 ± 0.8		18.0 ± 4.1	e

Table S3 Total* concentration (mg g^{-1} DM^{-1}) of selected elements in the substrate prior to fertilization. Values are means ± SD.

aluminum (Al)	17.84 ± 0.49
calcium (Ca)	18.47 ± 0.28
iron (Fe)	11.02 ± 0.33
potassium (K)	2.25 ± 0.11
magnesium (Mg)	2.05 ± 0.08
manganese (Mn)	0.12 ± 0.00
phosphorus (P)	0.71 ± 0.01

*digested in a microwave digestion system (CEM Mars5, CEM Corporation, Matthews, NC, USA) at 180 °C in 3 ml HNO_3, 1.5 ml HF and 3 ml H_3BO_3

Figure S2 Element concentration in grains and flag leaf blades of *Triticum aestivum* L. cv. Akteur (mg g^{-1} $AFDM^{-1}$, Si-0) at harvest. Values are means (bars) ± SD (whiskers).

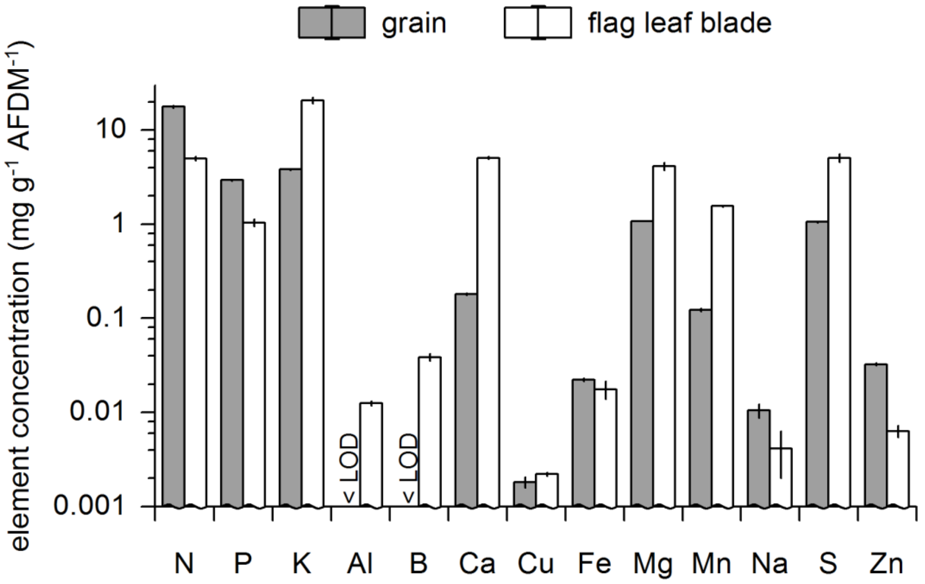

Supplement: Supplementary Information [file srep40829-s1.pdf]
